# Patients’ Dreams and Unreal Experiences During Intensive Care Unit (ICU) Hospitalization

**DOI:** 10.7759/cureus.51588

**Published:** 2024-01-03

**Authors:** Konstantina Dimou, Agapi L Batiridou, Fotios Tatsis, Spiros Georgakis, Zoe Konstanti, Georgios Papathanakos, Stefanos Mantzoukas, Elena Dragioti, Mary Gouva, Vasilios Koulouras

**Affiliations:** 1 Department of Nursing, School of Health Sciences, University of Ioannina, Ioannina, GRC; 2 Faculty of Medicine, School of Health Sciences, University of Ioannina, Ioannina, GRC; 3 Department of Intensive Care Unit, University Hospital of Ioannina, Ioannina, GRC

**Keywords:** memories, visions, unreal, dreams, critical care

## Abstract

In the intensive care unit (ICU), patients often experience fragmented memories, primarily comprising dreams and illusions. These experiences can impact psychosocial well-being, correlating with post-traumatic stress symptoms and heightened anxiety. Understanding these phenomena is crucial for holistic care. To systematically explore patients' perspectives concerning the recollection of dreams and unreal encounters during their stay in the ICU, considering pertinent clinical conditions and potential influencing factors, we conducted a comprehensive search in the PubMed/MEDLINE, Web of Science, and Scopus databases until November 20, 2023, following Preferred Reporting Items for Systematic Reviews and Meta-Analyses (PRISMA) guidelines. From an initial pool of 288 records, a thorough screening for eligibility resulted in the inclusion of nine studies for this systematic review. These selected studies underwent evaluation using either the Critical Appraisal Skills Programme (CASP) Qualitative Checklist or the Newcastle-Ottawa Scale (NOS). All studies categorized dreams into three main types: positive, distressing (including nightmares), and neutral experiences. These were further detailed based on aspects such as time, space, senses, emotions, and distinguishing between reality and unreality. Two studies found associations between dreams and conditions like Guillain-Barré syndrome (GBS), mental abnormalities, and delirium. In one study, GBS patients had more vivid dreams, hallucinations, and delusions compared to ICU control group patients; delirious patients tend to report more frequent frightening dreams. Patients in the ICU who recalled dreams often had more severe illness, longer stays, and higher ventilation frequency. Notably, a prolonged ICU stay significantly predicted the likelihood of dream recall, as consistently observed in three other studies. This suggests that patients with prolonged ICU stays, experiencing higher dream recall, underwent extended treatments. This systematic exploration of patients' perspectives on fragmented memories underscores the connections between these experiences, clinical conditions such as GBS and delirium, and extended ICU stays. Recognizing and attending to these psychological aspects in post-ICU care is critical for alleviating the enduring emotional consequences for patients.

## Introduction and background

The recollection of experiences by critically ill adult patients during their stay in the intensive care unit (ICU) has garnered substantial attention within pertinent literature over recent decades [1]. Existing research suggests that nearly half of these patients are unable to recollect their experiences [2]. Among those who do retain memories, their recollections often manifest as fragmented, primarily consisting of dreams, illusions, and so-called unreal experiences [1,3,4]. Furthermore, the capacity for recollection has demonstrated a correlation with the duration of ICU confinement [1].

The enduring implications of these experiences remain incompletely understood, while their prevalence has directly correlated with symptoms associated with post-traumatic stress disorder (PTSD), heightened anxiety, and a diminished quality of health-related life [1,5-7]. Longitudinal studies tracking ICU patients indicate that certain individuals vividly recall distressing memories such as being reliant on a ventilator, enduring pain, or undergoing nightmares. These recollections persist and can impede psychosocial functioning [8-10]. Moreover, the substance of dreams and experiences during ICU stays can serve as valuable insights for interpreting patients' unconscious emotions and meanings, thereby contributing to a holistic approach to their care [7].

While the occurrence, context, and recall of dreams and unreal experiences in the ICU, along with the factors associated with their manifestation, have been previously discussed in ICU literature, a comprehensive elucidation of these experiences and their contextual circumstances from the patients' viewpoint is lacking. Therefore, we aimed to systematically explore patients’ perspectives concerning the recollection of dreams and unreal experiences encountered during their ICU hospitalization, the pertinent clinical conditions, and potential factors related to the occurrence of these experiences.

## Review

Study design

This systematic literature review assessed the frequency and prevalence of dreams among patients in the ICU, while adhering to the Preferred Reporting Items for Systematic Reviews and Meta-Analyses (PRISMA) guidelines [11].

Search strategy

PubMed/MEDLINE, Web of Science, and Scopus databases were searched up to November 20, 2023, to identify studies reporting data on the dreams of patients in the ICU across all age groups. The search strategy included a database search using the terms "(ICU) AND (patients) AND ((dream) OR (vision))". We conducted a manual review of the cited references for each pertinent study identified during the search process. This was performed to ensure the inclusion of any additional eligible studies that might not have been captured through the electronic search.

Selection criteria

The systematic review was guided by the Population, Exposure, Outcome (PEO) framework to establish the study query. The target population encompassed hospitalized patients, with a focus on those within the ICU setting, while the primary outcomes centered on the frequency and prevalence of dreams. Eligible studies included those involving participants from general or community-based datasets. To ensure the reliability of findings, several exclusion criteria were implemented. Firstly, studies not directly addressing the subject of dreams were excluded. Secondly, those not specifically pertaining to the ICU were also omitted. Thirdly, studies published in languages other than English were excluded. Lastly, our study exclusively comprised patients who survived their ICU stay.

Data collection

All records gathered through the outlined search strategy were imported into the EndNote reference management software. The removal of duplicates relied on matching author names, publication years, titles, and abstracts. Two reviewers (KD, AB) conducted an initial screening of records by reviewing titles and abstracts independently. Subsequently, full texts of the retained records were evaluated for eligibility after excluding irrelevant references. In cases of disagreement, a third senior reviewer (FT) mediated and resolved discrepancies through discussion.

Data extraction

One reviewer (KD) was responsible for the initial extraction of data from eligible studies, which underwent a meticulous verification process by a second reviewer (AB). In instances of discrepancies, a discussion was facilitated involving a third senior reviewer (FT). The extracted data encompassed various components including author details, publication year, study design, geographical location, methodology employed, tools for measurement, sample characteristics, demographic data (such as average age and gender distribution), length of stay in the ICU (LOS), definitions attributed to dreams, and classifications pertaining to dream types.

Data synthesis

Given the anticipated methodological and conceptual variations among studies, conducting data synthesis through meta-analysis was not viable. Consequently, we employed a synthesis method devoid of meta-analyses, utilizing vote counting and detailing essential study characteristics (e.g., study design, bias assessment, etc.), as well as highlighting primary findings [12].

Study quality assessment

Primary studies were evaluated using the Critical Appraisal Skills Programme (CASP) Qualitative Checklist [13] or the Newcastle-Ottawa Scale (NOS) [14]. Based on their findings from the completed checklists, the two reviewers (KD, AB) independently judged each publication as low, good, or high using pre-determined cut points. Publications that did not represent the study quality as defined by the checklist were considered for exclusion.

Search results

**Figure 1:**
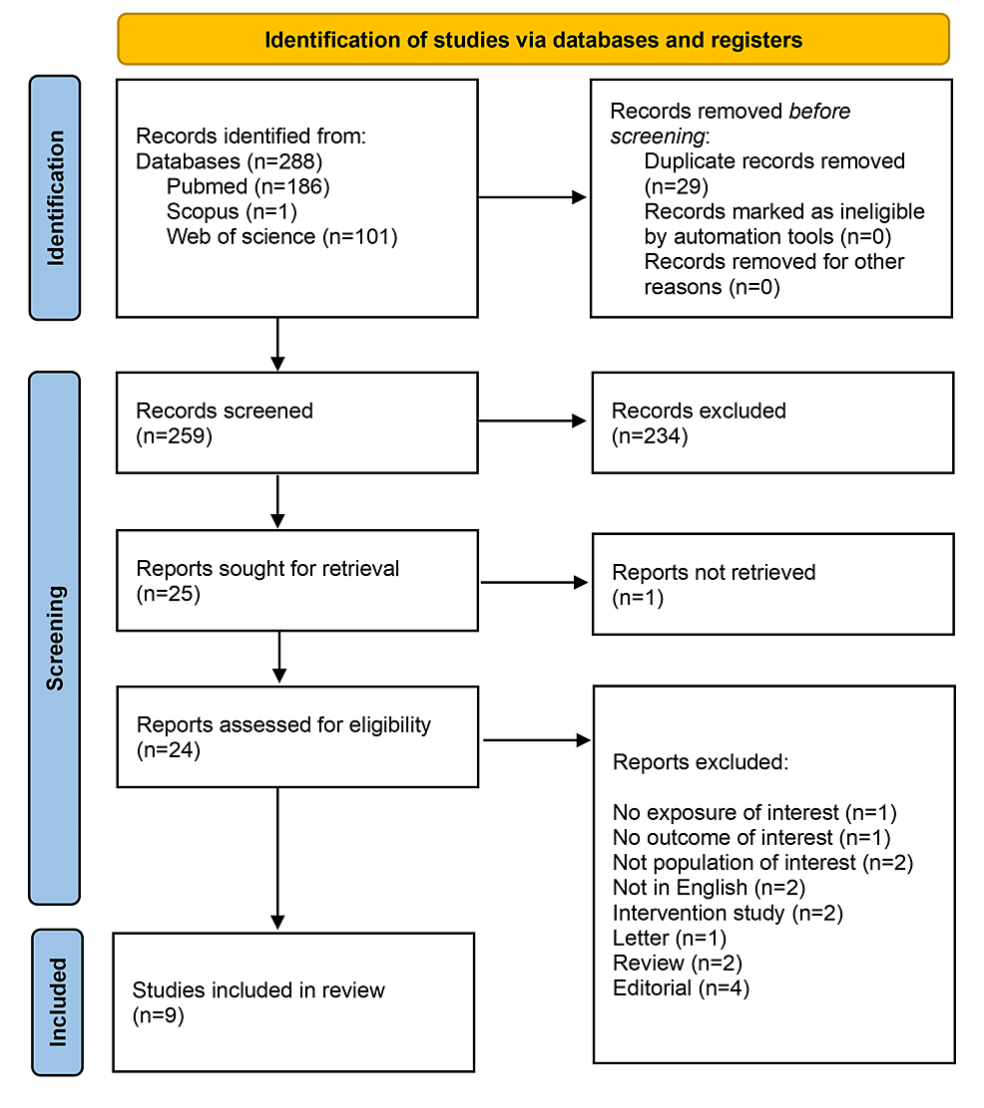
Study selection flowchart.

Through the electronic database search, a total of 288 potentially relevant records were initially identified. Following the elimination of duplicates, 259 distinct records underwent initial screening. Subsequently, 25 full-text articles were subjected to eligibility assessment, leading to the inclusion of nine individual publications in this systematic review (Figure 1). Among these, one report remained unattainable, and 15 publications were excluded for various reasons. These exclusions encompassed studies that did not report the designated exposure, lacked the specified outcome, did not involve the intended population, were in languages other than English, presented interventions, or were in the format of letters, reviews, or editorials.

Characteristics of included studies

The systematic review encompasses articles spanning from 1999 to 2023, comprising a diverse array of research methodologies: four qualitative investigations, three quantitative analyses, one cohort study, and one mixed-methods exploration, encompassing a total sample size of 642 individuals. These studies originate from multiple countries, including Germany, France, Greece, Italy, Sweden (with 2 studies), Australia (also with 2 studies), and one study conducted in Australia and New Zealand combined. Across the studies reporting this metric, the mean age of participants is reported at 50.81 years. The average percentage of male participants across the included studies is 55.52%, while the corresponding average for female participants is 44.48%. The duration of patients' stays in the intensive care unit ranged from 1 day to 3 weeks. Each individual study provided a distinct delineation of the definition and classification of dreams. The characteristics of the included studies are summarized in Table 1.

**Table 1:** General characteristics of included studies. N/R=Not Reported

Author, Year	Country	Study design	Methods of research (interview-questionnaire)	Measurement tools	Sample	Definition of dreams
Rundshagen et al., 2002 [1]	Germany	Clinical study	Structured interview	Demographic data, List of questions to evaluate patients’ memory, Simplified Acute Physiology Score (SAPS) II, Length of Stay in ICU, Mechanical ventilation, Sedation	n=289 [group 1=no recall (n=187), group 2=explicit recall (n=49), group 3=dreams/hallucinations (n=53)]	N/R
Papathanassoglou, & Patiraki, 2003 [7]	Greece	Qualitative study	Semi-structured with open-ended questions	Smith’s methodology (3 questions), Jung’s methodology (1 question)	n=8	N/R
Rapelli et al., 2023 [15]	Italy	Case study	Semi-structured interviews and self-report questionnaire & time points	Post-Traumatic Growth, Inventory (PTGI) Epworth Sleepiness Scale (ESS), Mannheim Dream Questionnaire (MADRE), Pittsburgh Sleep Quality Index (PSQI), Diary of sleep and dreams, The Depression Anxiety Stress Scale-21 (DASS-21), Imagery Rehearsal Therapy (IRT)	n=1	N/R
Granberg et al., 1999 [16]	Sweden	Qualitative study	Interview	hermeneutic approach	n=19	Unreal experiences
Roberts et al., 2006 [17]	Australia and New Zealand	Cohort study	Closed-ended and open-ended questions (interview)	Demographic data, Intensive Care Delirium Screening Checklist (ICDSC), Acute Physiology And Chronic Health Evaluation Score (APACHE II), Length of Stay in ICU	n=41 (delirious patients n=18, non-delirious patients n=23)	Unreal feelings, unreal sensations, factual memories
Cochen et al., 2005 [18]	France	Prospective controlled study	Interview/questionnaire	Demographic data, Clinical data (medical data), Data on mental status changes, Sleep monitoring	n=194 (patients with GBS n=139, patients without GBS n=55)	Vivid dreams (unusually clear, long dreams with an elaborate scenario and possibly strong emotions, that occurred only when sleeping and were acutely remembered), illusions (distortion of perceived images, sounds, or sensations), hallucinations (short-lasting perception without an object to be perceived, occurring either with open or closed eyes, in clear consciousness, delusions (the unreal perceptual experiences of non-confused patients that were long (day)-lasting, poorly criticized despite nurse’s intervention and caused observable behavioral changes
Löf et al., 2006 [3]	Sweden	Qualitative study	2 interviews (open questions)	N/R	n=9	Unreal experiences were classified as ‘‘hallucinations’’,‘‘nightmares’’, ‘‘strange dreams’’, ‘‘fantasies’’ and ‘‘delusions’’. Also, includes what patients remembered as disorientation and agitation (‘‘not acting or behaving normally’’)
Roberts & Chaboyer, 2004 [6]	Australia	Descriptive study	Semi-structured interviews	6 ethnographic questions (descriptive, contrast)	n=31 (n=23 reported dreams, n=8 did not report dreams	Unreal experiences are defined as visual and/or auditory phenomena, which appear in a condition experienced as totally wakeful or in a condition between wakefulness and sleep
Magarey & McCutcheon, 2005 [2]	Australia	Mixed methods study	Semi-structured interview, questionnaire (14 questions)	Semantic Differential Scale (SDS), Visual analogue scale	50 (only 8 participants had recalled dreams)	Nightmares, hallucinations, and confusion are all characteristics of the ‘ICU syndrome’

Methodological quality of included studies

Quality assessment for qualitative studies rated according to the number of criteria met: 1-4 = Low, 6-8 = Good, 9-10 = High [13]. Quantitative studies rated according to the number of criteria met: 1-3 = Low, 4-6 = Good, 7-8 = High [14]. All but one of the included publications received a quality assessment ranking of High (Tables 2, 3). It is important to note, however, that the variability in the study designs combined with the anecdotal nature of the reporting, constrained the authors' ability to rigorously evaluate bias specific to the construct. The decision to utilize two distinct appraisal tools was driven by the diverse nature of the included studies. This approach allowed for a comprehensive evaluation that aligned with the methodological nuances and strengths inherent in different study designs. By employing CASP for qualitative studies and NOS for quantitative studies, as described in the methods section, we aimed to ensure a comprehensive and contextualized assessment of the methodological quality across diverse research paradigms.

**Table 2:** Quality assessment based on the CASP qualitative research checklist. Y: yes; N: no; Critical Appraisal Skills Programme (CASP) criteria for qualitative studies: 1. Was there a clear statement of the aims of the research? 2. Was a qualitative methodology appropriate? 3. Was the research design appropriate to address the aims of the research? 4. Was the recruitment strategy appropriate to the aims of the research? 5. Was the data collected in a way that addressed the research issue? 6. Has the relationship between the researcher and participants been adequately considered? 7. Have ethical issues been considered? 8. Was the data analysis sufficiently rigorous? 9. Is there a clear statement of the findings? 10. (How valuable is the research?) *This is an open-ended question.

Author, Year	CASP checklist criteria										Quality
	1	2	3	4	5	6	7	8	9	10*	
Rapelli et al., 2023 [15]	Y	Y	Y	N	Y	Y	Y	Y	Y	(Y)	High
Granberg et al., 1999 [16]	Y	Y	Y	Y	Y	Y	Y	Y	Y	(Y)	High
Löf et al., 2006 [3]	Y	Y	Y	Y	Y	Y	Y	Y	Y	(Y)	High
Roberts & Chaboyer, 2004 [6]	Y	Y	Y	Y	Y	N	Y	Y	Y	(Y)	High
Magarey & McCutcheon, 2005 [2]	Y	Y	Y	Y	Y	N	Y	Y	Y	(Y)	High
Papathanassoglou & Patiraki, 2003 [7]	Y	Y	Y	Y	Y	Y	N	Y	Y	(Y)	High

**Table 3:** Quality assessment based on the Newcastle-Ottawa Scale. Each asterisk is equivalent to one point.

Author, Year	Newcastle-Ottawa Scale				
	Selection	Comparability	Outcome	Total Score	Result
Roberts et al., 2006 [17]	***	*	**	6	Good
Rundshagen et al., 2002 [1]	****	*	**	7	High
Cochen et al., 2005 [18]	***	*	**	6	Good

Definition of dreams

The majority of the reviewed articles (6 out of 9, constituting 66.6%) explicitly addressed the definition and conceptualization of dreams [2,3,6,16-18], while three of them opted to interchangeably employ the term “unreal experiences” in lieu of dreams [3,6,16]. Several terminologies were employed across the studies to encapsulate the spectrum of dream-related phenomena, including unreal feelings, unreal sensations, factual memories, vivid dreams, illusions, hallucinations, delusions, nightmares, and fantasies [2,3,6,16-18]. Specifically, Roberts and Chaboyer (2004), characterized dreams as “visual and/or auditory phenomena manifesting during a state perceived as either entirely awake or during the transitional phase between wakefulness and sleep" [6]. Furthermore, Cochen et al. (2005) expounded on various definitions, delineating vivid dreams as unusually lucid, extensive dream sequences often entailing elaborate scenarios and potentially intense emotions, exclusively occurring during sleep and being distinctly recalled [18]. Additionally, the above study discerned illusions as distortions of perceived sensory stimuli, hallucinations as transient perceptions lacking a corresponding external stimulus, occurring irrespective of eye state and during conscious awareness, and delusions as enduring, non-confused patients' unreal perceptual experiences typically persisting over extended periods (days), resisting critique despite nursing intervention, and precipitating observable behavioral alterations [18].

Types of dreams

All the studies examined in this context delineated three primary classifications of dreams: a) positive or pleasant, b) distressing or frightening, encompassing nightmares or experiences described as terrifying, persecutory, horrific, or frightening, and c) neutral or miscellaneous dream experiences [1-3,6,7,15-18]. Moreover, some of the authors provided detailed categorizations of dream types based on various attributes such as temporal aspects, spatial characteristics, auditory or visual elements, bodily sensations, perception alterations, feelings of solitude, spiritual themes, concepts of mortality and birth, distinctions between reality and unreality, color associations, feelings of powerlessness, and perceived purpose [2,7,16,17]. 

Dreams and conditions

Two studies within this context established correlations between dreams and specific conditions such as Guillain-Barre syndrome (GBS), mental abnormalities, and delirium [17,18]. One study presented the correlation between dreams, GBS, and mental abnormalities [18]. According to Cochen et al. (2005), patients with GBS had vivid dreams (5.7%), illusions (9%), hallucinations (18.6%, significant difference between GBS and ICU control group), and delusions (21.6%, significant difference between GBS and ICU control group) [18]. Moreover, eight patients who suffered both from GBS and mental abnormalities presented vivid dreams with strange, colorful, and highly emotional content (19%). Additionally, 13 patients of the same group were aware of visual, tactile, and auditory illusions (30%), 26 patients experienced hallucinations that mostly included colorful and moving figures or creatures (60%), and 30 patients presented paranoid delusions with abnormal behaviors that stemmed from a dream-like state (70%). On the contrary, patients in the ICU without GBS (control group) had less detailed vivid dreams (6/9, 66%) including nightmares about murder, war, insects, and two pleasant dreams of parents and traveling to China. In total, hallucinations were mostly experienced by 37% of ICU patients versus 17% of non-ICU patients (p= 0.02) [18].

Regarding the link between dreams and delirium, only one study explored this association. Roberts et al. (2006) noted that dreaming appeared to be more common among delirious patients (9 out of 18, 50%) compared to non-delirious patients (9 out of 23, 39%) [17]. However, statistical analysis did not show a significant correlation (Chi-square= 0.48, p= 0.49). Notably, only delirious patients exhibited dream recollections. Among the total 41 patients examined, 20 (49%) had factual memories, 14 (34%) had dreams along with memories, four (10%) solely recalled dreams, and three (7%) had no memory of their dreams. Moreover, employing simple logistic regression, the study found no substantial association between delirium status and dream recall (OR 1.56, 95% CI 0.45-5.41, p = 0.49). Furthermore, while most dreams were categorized as positive or neutral, delirious patients reported experiencing scary dreams more frequently than non-delirious patients [17].

Dreams and ICU stay

Four of the included studies presented a positive correlation between dreams and ICU stay [1,2,6,17]. The research conducted by Roberts et al. (2006) highlighted that individuals in the ICU who were able to recall dreams tended to exhibit more severe illness, extended ICU stays and were more frequently ventilated, particularly among female patients, compared to those who did not recall any dreams [17]. Additionally, the authors employed forward-stepwise logistic regression analysis involving significant and nearly significant variables, such as Length of Stay (LOS), Acute Physiology and Chronic Health Evaluation (APACHE II) score, and ventilation status. They determined that only the ICU LOS (OR 1.39, 95% CI 1.08-1.79, p= 0.01) emerged as a significant predictor for recalling dreams among ICU patients [17]. Similar observations were made in three other studies, indicating that patients recalling dreams or having a higher likelihood of dream recall tended to have prolonged ICU stays, necessitating extended assisted ventilation, sedation, and overall intensive care treatment [1,2,6]. Specifically, a clinical study delineated that among 162 patients, 32 individuals (19.8%) who had an ICU stay duration of less than one day reported experiencing dreams, encompassing four dreams (2.5%) categorized as nightmares, one (0.6%) characterized as a hallucination, and seven (4.4%) classified as miscellaneous dreams. Conversely, among 127 patients who stayed in the ICU for more than one day, 49 patients (38.6%) recounted having dreams, comprising 23 (18.1%) identified as nightmares, 18 (14.2%) classified as hallucinations, and eight (6.3%) categorized as miscellaneous dreams [1].

Dreams and age characteristics 

In a study conducted by Rundshagen et al. (2002), associations between gender and age characteristics were observed. The research indicated that younger patients exhibited a higher propensity for experiencing recall or dreams [1].

Discussion

In this systematic review, we aimed to synthesize ICU survivors’ scenes towards dreams and unreal experiences that occurred to them during their hospitalization in the ICU. Furthermore, our aim was to consolidate information on the surrounding clinical conditions and possible factors related to the occurrence of these experiences. Understanding ICU survivors' experiences of dreams and unreal sensations could significantly contribute to holistic patient care, psychological well-being, and the development of effective post-ICU support mechanisms [19-24].

We found that in most of the included studies, participants used the term “dreams” to describe their experiences. The terms unreal feeling, unreal sensation, factual memories, vivid dreams, illusions, hallucinations, delusions, nightmares, and fantasies were most commonly used and were directly related to dreams. More specifically, in Australia and New Zealand, Roberts et al. (2006) observed that the context of dreams included a state of sleep or hallucinations, an unreal feeling or sensation, or some delusional events [17]. The popularity of such vivid descriptors points towards a complex psychological state experienced by these patients [19].

The terms hallucinations and delusions were also used in both the German study by Rundshagen et al. (2002) and the French study conducted by Cochen et al. (2005) [1,18]. In Cochen et al. (2005), hallucinations were described as a short-lasting perception without an object to be perceived, occurring either with open or closed eyes, in clear consciousness, and delusions as an unreal perceptual experience of non-confused patients that were long (day)-lasting [18]. In Rundshagen et al. (2002) hallucinations and delusions included mostly unpleasant experiences [1]. Other terms like vivid dreams, were also observed and described as acutely remembered long lasting, clear dreams with specific scenarios that caused strong emotions [18]. In the included study conducted by Magarey and McCutcheon (2005) in Australia participants reported hallucinations, delusions, and nightmares [2].

Unreal experiences were defined in the relevant included study by Roberts and Chaboyer (2004) in Australia [6], as visual and/or auditory phenomena, that appear in a condition experienced as totally wakeful or in a condition between wakefulness and sleep and were classified as ‘‘hallucinations’ ’nightmares’’, ‘‘strange dreams’’, ‘‘fantasies’’ and ‘‘delusions’’ by Löf et al. (2006) [3]. Granberg et al. (1999) observed that participants also used the term unreal experiences to describe dreams, nightmares, stupid fantasies, crazy dreams, fantasies, changes of perspective, illusions, or dreamlike experiences [16].

Most of the above-mentioned experiences were found to be disturbing and described as “scary”, ‘‘terrifying’’, ‘‘really horrible’’ or ‘‘frightening’’ [3,6,18]. However, when they were described as pleasant the term used was “dreams” [3]. The predominance of disturbing, frightening, or terrifying experiences, as opposed to pleasant or positive ones, suggests a psychological landscape that is often marked by distress, fear, and emotional turmoil. Patients undergoing these negative experiences might encounter significant psychological distress, potentially leading to heightened anxiety, stress, or even post-traumatic stress disorder (PTSD)-related symptoms [1,6].

The occurrence of these experiences was associated with some clinical conditions, ICU length of stay, and age. Roberts et al. (2006) in Australia and New Zealand observed that delirious patients were more likely to recall dreams that occurred during their ICU stay and patients with GBS were found by Cochen et al. (2005) to experience and recall vivid dreams and illusions, hallucinations, and paranoid delusions more frequently than patients without GBS [17,18]. These findings underscore the correlation between certain medical conditions and the heightened occurrence of recalling vivid and often distressing dream-like experiences in the ICU environment.

Some studies [1,2,6,17] also identified that the occurrence and recollection of dreams and unreal experiences were directly associated with the length of ICU hospitalization as patients who remembered dreams stayed longer in the ICU [1,6]. These associations further underscore the psychological intricacies at play. The duration of critical care treatment might play a role in the manifestation or recollection of these experiences, emphasizing a potential correlation between prolonged ICU stays and the occurrence of vivid and sometimes disturbing dreams or unreal perceptions. Patients who stayed longer in the ICU or were younger were more prone to experiencing and recalling dreams and unreal experiences [1]. This implies a potential vulnerability among these patient groups to encounter distressing psychological phenomena during their ICU stay.

The psychological impact of these experiences extends beyond the ICU setting, affecting the quality of life for survivors. Traumatic experiences, particularly those characterized by fear or unpleasantness, have the potential to leave a lasting psychological impact, contributing to post-ICU psychological distress and complicating the recovery process [1,6]. However, psychological support and repeated detailed information are observed to offer a therapeutic effect [25]. Psychological support mechanisms, such as counseling, psychotherapy, or interventions provided by mental health professionals, have shown promising results in alleviating the emotional burden caused by these vivid and often disturbing dreams, hallucinations, or unreal perceptions [26-30]. Providing also comprehensive explanations about the nature of these dreams or unreal experiences, their possible causes, and reassurance about their transient and non-harmful nature could contribute significantly to reducing anxiety and fear associated with these encounters [25].

This systematic review offers a detailed exploration of ICU survivors' perspectives on dreams, hallucinations, and unreal experiences during their hospitalization, highlighting their impact on patient's emotional well-being and the need for tailored post-ICU care. The inclusion of studies from various countries like Australia, New Zealand, France, and Germany provides a multinational perspective, contributing to a broader understanding of these experiences across different cultural backgrounds. In addition, we thoroughly investigated and analyzed the various words and expressions employed by individuals who underwent ICU experiences to articulate and convey their encounters.

This review reveals the profound and far-reaching implications of ICU survivors' experiences, highlighting a prevalence of distressing dream-like encounters that contribute to a psychological landscape marked by distress, fear, and emotional turmoil. The study emphasizes the need for a nuanced understanding of diverse terms like hallucinations, vivid dreams, and unreal sensations used to describe these experiences. Associations between medical conditions, ICU length of stay, and age underscore a vulnerability in specific patient groups, particularly among those who stayed longer in the ICU or were younger, suggesting an increased likelihood of encountering distressing psychological phenomena during their ICU stay. The psychological impact extends beyond the ICU, affecting survivors' quality of life, with traumatic experiences characterized by fear potentially leading to lasting psychological effects. However, the study highlights the therapeutic effect of psychological support mechanisms, such as counseling and psychotherapy, in alleviating the emotional burden caused by vivid and disturbing dreams. The multinational perspective enriches the understanding of these experiences across diverse cultural backgrounds.

Despite the study's strengths, there are specific shortcomings or constraints that need to be acknowledged. The diverse terms employed by participants to describe their experiences across studies might introduce variations and ambiguities, complicating comparisons, and generalizations. Although associations between clinical conditions, ICU length of stay, and the occurrence of experiences were observed, establishing causality remains challenging due to the nature of observational studies included in the review. While the review predominantly examines disturbing and frightening experiences, it may lack emphasis on the spectrum of positive or neutral encounters, potentially limiting a holistic understanding of these occurrences. Future research would benefit from standardized terminologies and deeper insights into positive or neutral experiences to provide a more comprehensive perspective in this regard. Finally, we encountered limitations in identifying adequate literature or studies that specifically focused on family members' experiences or perspectives regarding dreams and unreal sensations. The lack of available research on this aspect restricts our ability to provide a comprehensive overview or inclusion of family-related perspectives in our study's conclusions.

## Conclusions

In conclusion, this systematic review delved into the multifaceted field of ICU survivors' perceptions, particularly focusing on the vivid dreams, hallucinations, and unreal experiences that manifested during their hospitalization. The terminology used by participants, such as dreams, hallucinations, delusions, and vivid dreams, varied across studies, emphasizing the diverse nature of these encounters. These experiences, often characterized as disturbing or frightening, were linked to clinical conditions, ICU length of stay, and age. Notably, patients with delirium and specific medical conditions like Guillain-Barré syndrome exhibited a higher likelihood of recalling intense dreams and unreal sensations. The association with ICU length of stay suggests a potential correlation between the duration of critical care treatment and the occurrence of these experiences. Younger age was also identified as a factor influencing the frequency of dreams and unreal experiences.

Furthermore, the emotional impact of these experiences extends beyond the ICU, affecting the quality of life for survivors. Traumatic in nature, these encounters, especially those with unpleasant content, contribute to anxiety and PTSD-related symptoms. On a positive note, psychological support and detailed information dissemination emerged as therapeutic interventions, underscoring the importance of post-ICU care and addressing the psychological aftermath of such experiences. Overall, understanding the diverse range of these occurrences and their impact on patients is crucial for developing targeted interventions and support strategies for ICU survivors.
